# Development of a Monoclonal Antibody Specific to the IgM Heavy Chain of Bighead Catfish (*Clarias macrocephalus*): A Biomolecular Tool for the Detection and Quantification of IgM Molecules and IgM^+^ Cells in *Clarias* Catfish

**DOI:** 10.3390/biom10040567

**Published:** 2020-04-07

**Authors:** Anurak Bunnoy, Uthairat Na-Nakorn, Prapansak Srisapoome

**Affiliations:** 1 Laboratory of Aquatic Animal Health Management, Department of Aquaculture, Faculty of Fisheries, Kasetsart University, 50 Paholayothin Rd, Ladyao, Chatuchak, Bangkok 10900, Thailand; anurak.bunnoy@gmail.com; 2 Laboratory of Aquatic Animal Genetics, Department of Aquaculture, Faculty of Fisheries, Kasetsart University, 50 Paholayothin Rd, Ladyao, Chatuchak, Bangkok 10900, Thailand; ffisurn@ku.ac.th

**Keywords:** monoclonal antibody, IgM heavy chain, catfish, ELISA, flow cytometry, B cells

## Abstract

Catfish is a commonly-cultivated freshwater fish in Thailand and many Southeast Asian countries. The molecular data obtained for the IgM heavy chain (IgMH) of catfish have been useful for distinguishing monoclonal antibodies (mAbs). A mAb specific to Cμ_1_ of the IgMH of catfish (IgMHCμ_1_ mAb) was developed in a rabbit model using sequence information from bighead catfish (*Clarias macrocephalus*). The IgMHCμ_1_ mAb strongly recognized the IgM heavy chain of the tested catfish, namely, bighead catfish, African catfish (*Clarias gariepinus*) and their hybrid (*C. macrocephalus* × *C. gariepinus*), in immunological Western blot analysis and competitive ELISAs. Additionally, the IgMHCμ_1_ mAb successfully recognized IgM^+^ cells by detecting IgM molecules in both secreted and membrane-bound forms in peripheral blood leukocytes (PBLs). The IgMHCμ_1_ mAb was further used to quantify the percentage of IgM^+^ cells among PBLs through flow cytophotometry. The IgM^+^ cell percentages of healthy bighead catfish, African catfish and their hybrid were 38.0–39.9%, 45.6–53.2%, and 58.7–60.0%, respectively. Furthermore, the IgMHCμ_1_ mAb showed no cross-reactivity with the IgM of zebrafish. These findings suggest that this mAb can be used as an immunological tool for monitoring the health, immune status, and immune development of cultivated *Clarias* catfish.

## 1. Introduction

The catfish industry is the second largest long-lasting freshwater fish aquaculture industry in Thailand after the Nile tilapia (*Oreochromis niloticus*) industry. The total production of catfish farming increased more than twofold from 76,000 million tons in 2000 to 159,314 million tons in 2004 (FAO, 2017) [1]. Among the catfish species found in Thailand, the bighead catfish (*Clarias macrocephalus* Gunther, 1864) is a well-known species with economically important characteristics, especially special meat flavors and textures [2,3]. Unfortunately, this fish exhibits natural limitations under many conditions. Bighead catfish are slow growing, defective in fry production, susceptible to infectious disease, and sensitive to various environmental conditions [4]. These constraints strongly affect catfish production in Thailand [5,6,7]. As an alternative, the production of hybrid catfish (female bighead catfish, *C. macrocephalus* x male African catfish, *C. gariepinus*) has attracted attention in the catfish industry in Thailand and Southeast Asia. Hybrid catfish are known as “big-aui” in Thai [7]. More specifically, hybrid catfish acquire major commercially-desirable characteristics from their parents, such as good meat quality, rapid growth, improved feed conversion, increased survival, resistance to many diseases, and tolerance to many environmental conditions. Therefore, bighead catfish are increasingly being used for hybrid catfish production in Thailand [2,3,4,5,6,7].

The industry is experiencing extensive demand, increased output of catfish, the development of intensive catfish aquaculture systems, and rapid global environmental changes. Intensively-farmed fish are occasionally subjected to stressful conditions, which can lead to outbreaks of diseases caused by specific pathogenic bacteria, especially *Aeromonas hydrophila* and *Flavobacterium columnare* [8,9], which are difficult to handle and difficult to predict. Consequently, studies of the catfish immune system could provide a better understanding of catfish immunity and scientific strategies for effective and efficient health management measures during catfish cultivation.

Based on current information, there are two major immune response levels in teleosts, namely, the nonspecific (innate) immune response and specific (acquired) immune responses [10,11]. The innate immune system exhibits a fast, nonspecific response to the pathogen infecting the host organism, while the adaptive immune system responds to a particular pathogen with highly discriminatory, specific, memory-based, and long-lasting immune responses [12,13]. B lymphocytes are crucial orchestrated lymphocytes in the adaptive immune system of vertebrates [11,12,13].

**Figure 1:**
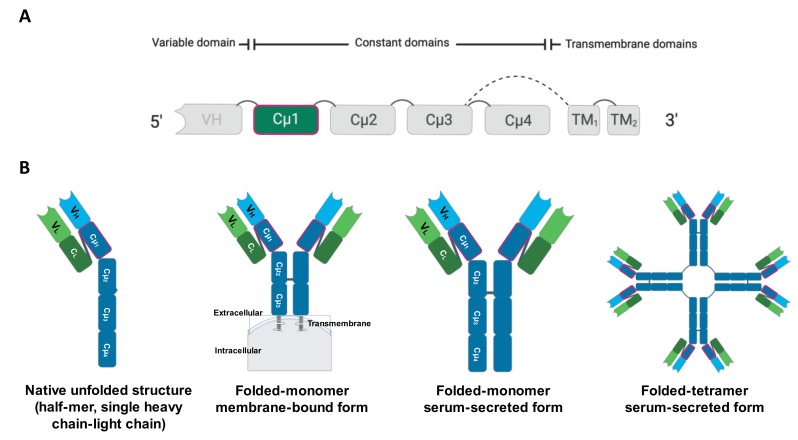
General molecular structure and alternative splicing pattern of the IgM heavy chain in teleost fish (**A**). Comparison among teleost immunoglobulin isoforms, which are generally expressed in membrane-bound form (B cell receptor) or as a secreted native unfolded-half-mer structure, folded-monomer, or folded-tetramer in the serum (**B**).

IgM is a predominant B cell product that is considered the primitive Ig class identified in teleost fish. It can be secreted or expressed on the membrane surface of B cells. Secreted folded-monomeric or folded-tetrameric IgM constitutes the main serum Ig in fish [14,15]. In contrast, the membrane-bound form is smaller and contains a transmembrane exon at the C-terminus (Figure 1) [11,15]. Functionally, Igs specifically recognize and bind to a particular antigen to conduct various immune effector defensive processes, including neutralization, opsonization, antibody-dependent cell-mediated cytotoxicity (ADCC), and complement activation [16].

In recent decades, molecular studies of the Ig gene in catfish have been important in both basic and applied research. Their applications are especially useful in fish cultivation [17,18], in pathogen detection where they provide diagnostic reagents, and for therapy [19,20,21]. Furthermore, such studies can facilitate improving the aquaculture of catfish by selecting for specific disease-resistance traits.

This study examined the molecular structure of the IgM heavy chain in these catfish in great detail, with a particular focus on their most highly-conserved regions of the IgM protein. The recombinant protein and monoclonal antibodies (mAbs) specific to the IgM heavy chain were developed and produced. In addition, the applications of the obtained mAbs were further investigated by immunoassay techniques, including enzyme-linked immunosorbent assays (ELISAs), Western blotting, indirect immunofluorescent assay tests (IIFATs), and flow cytometric analysis of specific lymphocytes of catfish. This research was performed using new immunological tools, which are believed to be beneficial for sustainable catfish aquaculture at the global level.

## 2. Results

### 2.1. Molecular Cloning, Characterization, and Antigenicity Analysis of the IgMHCμ_1_ Sequence of Bighead Catfish

**Figure 2:**
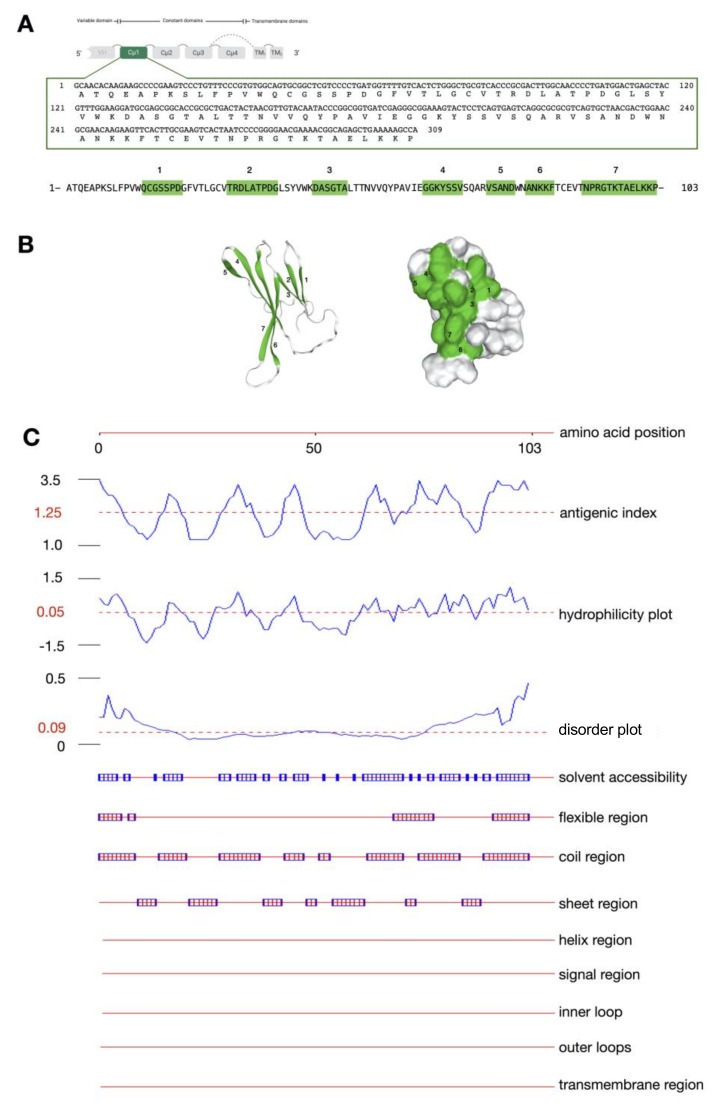
Nucleotide and amino acid sequences for the Cμ_1_ protein of the IgM heavy chain of bighead catfish (**A**), amino acid sequence and schematic structures indicate the antigenic determinant regions located in the Cμ_1_ protein (highlighted in green) (**B**), and in silico analysis of the immunogenetic determinant property of the Cμ_1_ protein of the IgM heavy chain of bighead catfish (**C**).

The IgMH cDNA sequence encoding the Cμ_1_ protein was successfully cloned into the pET28b(+) expression vector. The nucleotide sequence length of Cμ_1_ of bighead catfish was 309 bp, which could be translated to 103 amino acid residues. The molecular weight and p*I* were approximately 11.01 kDa and 8.52, respectively, (Figure 2A). No *N*-linked glycosylation was observed in this sequence. Furthermore, the antigenicity of the IgMH sequence encoding the Cμ_1_ protein was located in seven different positions of Cμ_1_: QCGSSPD, TRDLATPDG, DASGTA, GGKYSSV, VSAND, ANKKF, and NPRGTKTAELKKP (Figure 2B). The Cμ_1_ protein is most likely a hydrophilic and flexible molecule. In addition, sequence features such as the helix region, signal region, inner loop, outer loop, and transmembrane region were not found in the Cμ_1_ protein sequence by in silico analysis (Figure 2C).

### 2.2. Expression of the IgMHCμ_1_ Recombinant Protein

**Figure 3:**
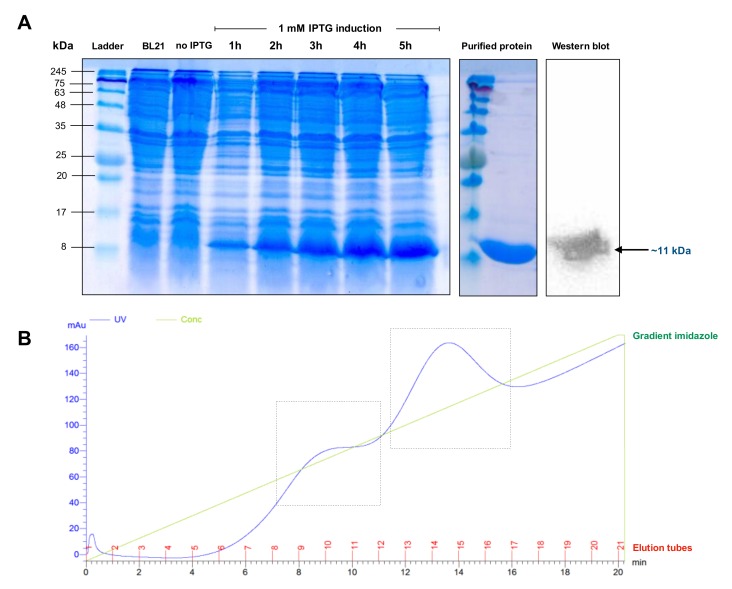
Overexpression, purification, and Western blot analysis of the Cμ_1_ protein of the IgM heavy chain of bighead catfish (**A**) and chromatogram for purification of the Cμ_1_ protein using Ni-NTA affinity in different continuous linear gradients of imidazole (**B**).

The IgMH Cμ_1_ protein was overexpressed in a bacterial expression system using *E. coli* BL21(DE3). Sodium dodecyl sulfate-polyacrylamide gel electrophoresis (SDS-PAGE) revealed that the Cμ_1_ protein was well expressed after isopropylthio-β-galactoside (IPTG) induction from the first hour of induction, with a distinct band at approximately 11 kDa (Figure 3A). This result is consistent with the predicted molecular weight as previously described. High purity of the Cμ_1_ protein was obtained after purification with the HiTrapTM Chelating HP system (Figure 3A,B).

### 2.3. Production of the IgMHCμ_1_ mAb against IgMH in Catfish

The IgMHCμ_1_ mAb exhibited good immunogenicity in rabbits, and the increase in the serum antibody titer was approximately >1:512,000 at day 60 after immunization. The yield of purified IgMHCμ_1_ mAb obtained from hybridoma cells was 5.86 mg, with purity > 90%.

### 2.4. Characterization and Application of the IgMHCμ_1_ mAb against IgMH in Catfish

#### 2.4.1. Establishment and c-ELISA Analysis

**Figure 4:**
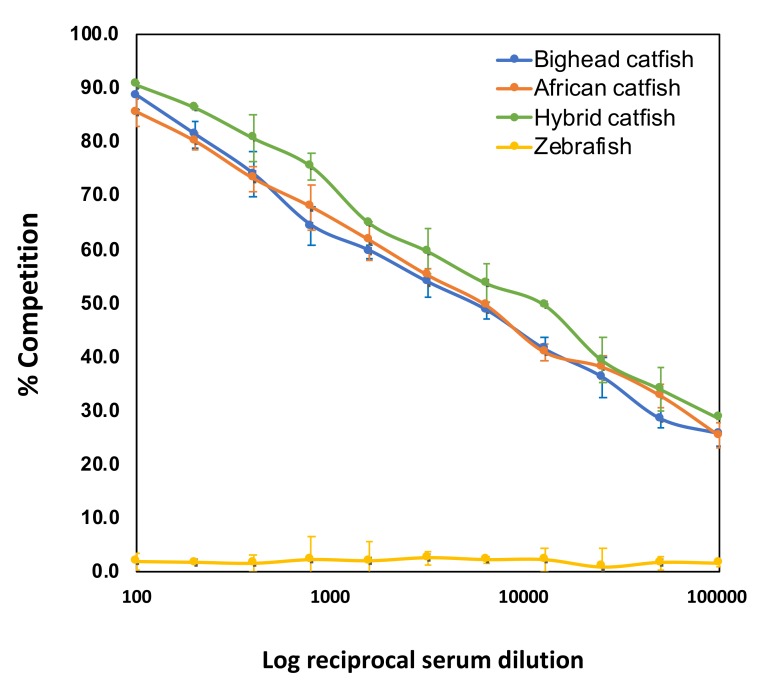
Specificity of the antigenic relatedness of the monoclonal antibody (mAb) specific to Cμ_1_ of the IgM heavy chain (IgMH) (IgMHCμ_1_ mAb) within the catfish genus *Clarias* and between distinct species (zebrafish), as determined by indirect competition ELISA.

The new IgMHCμ_1_ mAb of bighead catfish was optimized for the prior ELISA using standard checkerboard titration (CBT). The optimum concentration of purified antigen CH_1_ protein and serum dilution of the sample were 32 ng/mL/well and 1:320, respectively. Furthermore, the optimum dilutions of the IgMHCμ_1_ mAb and secondary HRP-conjugated goat anti-rabbit IgG were 1:51,200 (20 ng/mL/well) and 1:12,400 (40 ng/mL/well), respectively. The c-ELISA results revealed that the IgMHCμ_1_ mAb was highly specific to all tested catfish, including bighead catfish, African catfish, and hybrid catfish, at all tested serum dilutions. Furthermore, no competition was observed in zebrafish at any serum dilution (Figure 4).

#### 2.4.2. Western Blot Analysis

**Figure 5:**
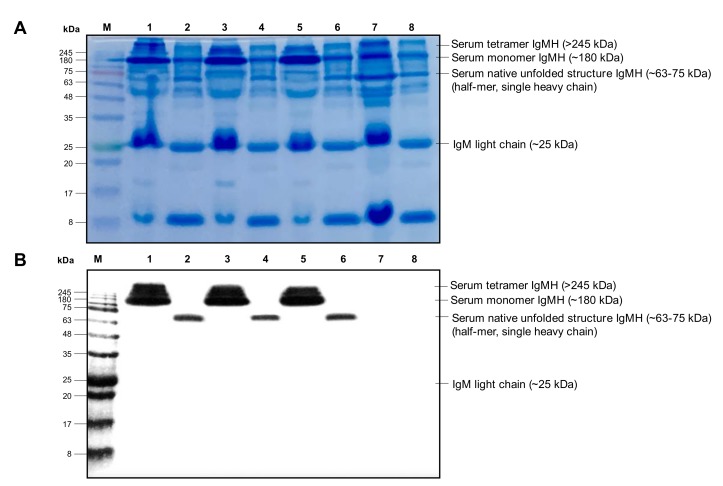
(**A**) Sodium dodecyl sulfate-polyacrylamide gel electrophoresis (SDS-PAGE) analysis of catfish fish serum under native condition (lanes 1, 3, 5, and 7) and reducing conditions (lanes 2, 4, 6, and 8) and (**B**) Western blot analysis of the IgMHCμ_1_ mAb detects the serum tetramer, monomer IgMH and serum native unfolded-halfmer molecules in catfish and zebrafish. M, protein marker (kDa); lanes 1 and 2, bighead catfish; lanes 3 and 4, African catfish; lanes 5 and 6, hybrid catfish; and lanes 7 and 8, zebrafish.

The results of Western blot analysis under native and reducing conditions showed that the IgMHCμ_1_ mAb of bighead catfish reacted strongly with the serum tetramer, monomer, and serum native unfolded-halfmer IgMH molecules in catfish. This mAb exhibited cross-reactivity in closely related catfish, that is, African catfish and hybrid catfish, recognizing >245, ~180 and ~63–75 kDa for the serum tetramer, serum monomer, and serum native unfolded-halfmer IgMH molecules, respectively. In addition, detection of the serum IgM heavy chain by the Western blot assay was not observed in zebrafish, which was further used as a negative control fish sample (Figure 5).

#### 2.4.3. IIFAT for the Detection of IgM^+^ Cells

**Figure 6:**
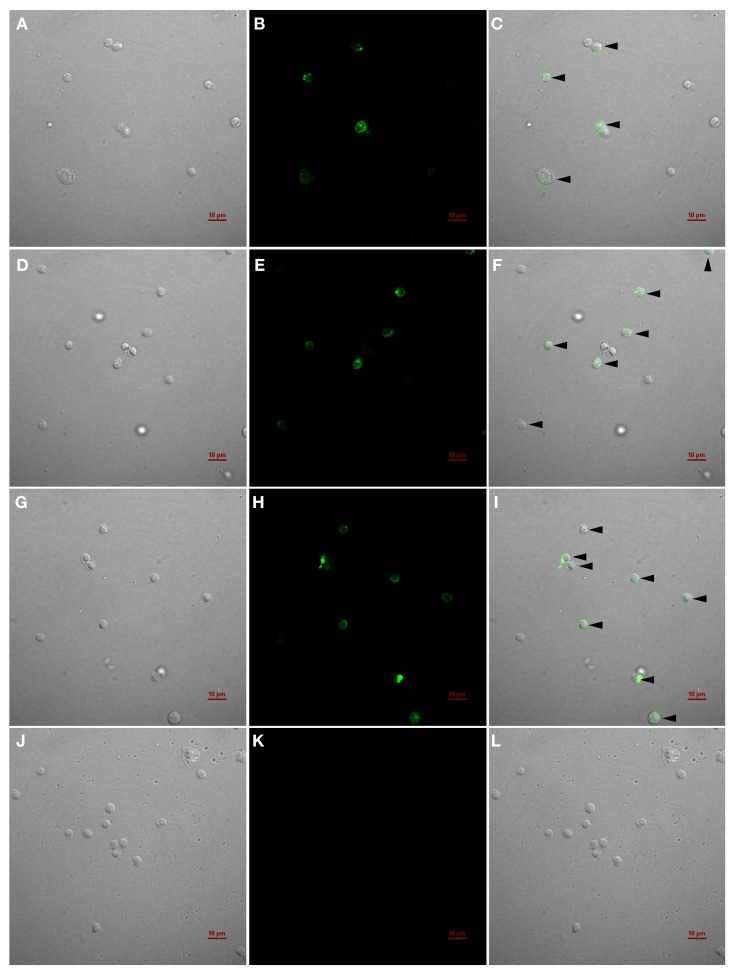
Indirect immunofluorescence analysis of the IgMHCμ_1_ mAb by detecting IgM^+^ cells (black arrows) in peripheral blood leukocytes (PBLs) of bighead catfish (**A–C**), African catfish (**D–F**), hybrid catfish (**G–I**) and zebrafish (**J**–**L**).

The detection of IgM^+^ cells using mAb IgMHCμ_1_ through the IIFAT revealed specific fluorescence signals in the intracellular regions and on the cell membranes (Figure 6) of healthy bighead catfish (Figure 6A–C), African catfish (Figure 6D–F), and hybrid catfish (Figure 6G–I). The results indicate that the IgMHCμ_1_ mAb could specifically recognize both intracellular and membrane-bound forms of IgM molecules in lymphocytes. No reactivity of mAb IgMHCμ_1_ was found in the PBLs of zebrafish (Figure 6J–L).

#### 2.4.4. Flow Cytometry for the Quantification of IgM^+^ Cells

**Figure 7:**
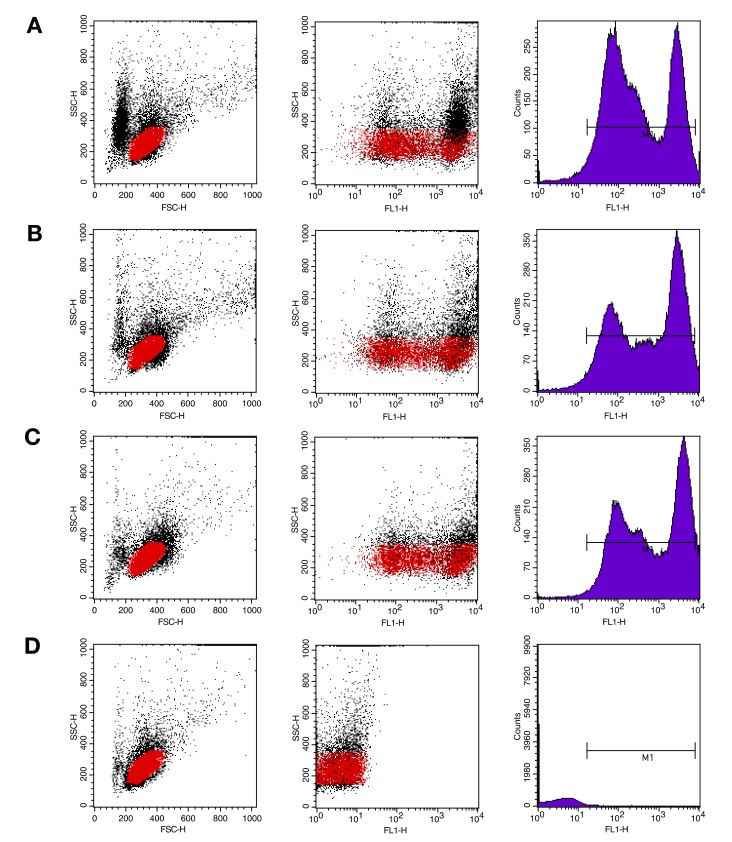
Flow cytometric analysis of the IgMHCμ_1_ mAb by quantification of IgM^+^ cells in the PBLs of bighead catfish (**A**), African catfish (**B**), hybrid catfish (**C**), and zebrafish (**D**).

The IgMHCμ_1_ mAb was then used to quantify the IgM^+^ cell population in the PBLs of healthy catfish by flow cytometry. The results showed that the percentages of IgM^+^ cells in the PBLs of healthy bighead catfish (Figure 7A), African catfish (Figure 7B), and hybrid catfish (Figure 7C) were 38.0–39.9%, 45.6–53.2%, and 58.7–60.0%, respectively (Figure 7A–C). The IgMHCμ_1_ mAb was not used for recognizing the IgM^+^ cells in the PBLs of zebrafish (Figure 7D). The percentages of IgM^+^ cells in PBLs of teleost fish determined using mAb are compared in Table 1.

## 3. Discussion

Studies on the fish immune system have been rapidly increasing worldwide for many years to obtain a complete understanding of fish immunity, which is closely related to immunity in most mammals. Thus, the study of immunity in catfish has important implications in basic sciences and applications in catfish cultivation. In teleosts, three different Ig heavy chain isotypes have been identified, namely, IgM, IgD, and IgT/Z. Not every class is expressed in each fish species studied. IgM is found in all gnathostome vertebrate species, IgD is also widely distributed among vertebrates, and IgT/Z (for teleosts/zebrafish) is specific to teleost fish. The fish IgM, D, and T/Z classes refer to the protein products of the isotypes μ, δ, and τ/ζ, respectively, which correspond to their associated constant genes [10,11,26]. Over the past decade, in this new era of immunological research, monoclonal antibodies (mAbs) have been recognized as a novel technology that can be beneficial tools for the study of catfish; mAbs can be used as cell-specific markers, for the quantification of target cells in immunoassays, as diagnostic reagents in pathogen detection, and for therapy [19,20,21]. Consequently, mAbs specific to IgM molecules have been developed and produced for determining health status in various important economic fish species, including common carp (*Cyprinus carpio*), channel catfish (*Ictalurus punctatus*), cod (*Gadus morhua*), eel (*Anguilla Anguilla*), Japanese flounder (*Paralichthys olivaceus*), red drum (*Sciaenops ocellatus*), Atlantic salmon (*Salmo salar*), sea bream (*Sparus aurata*), sea bass (*Dicentrachus labrax*), rainbow trout (*Oncorhynchus mykiss*), and turbot (*Scophthalmus maximus*) [10,11,26,27,28,29,30,31,32,33]. Meanwhile, information on specific immunity and its application in catfish in the genus *Clarias* have never been reported to date. This is the first report of mAb development based on the knowledge of *Clarias* catfish immunity.

At the cellular level of the immune response, the antigenic determinants or epitopes of antigens play a pivotal role in the immune response, which is activated through B cells and T cells [34,35]. In this study, the IgMHCμ_1_ mAb was developed with specificity for the constant Cμ_1_ domain of the IgM heavy chain of bighead catfish. The Cμ_1_ of the IgM protein in vertebrates is considered the most primitive and highly-conserved exon among the Ig molecules [11,14]. In silico sequence analysis of Cμ_1_ revealed seven proteomic features that are associated with antigenicity on the Cμ_1_ exon. A recent study suggested that the antigens contain multiple antigenic determinants or epitopes with the potential to trigger the activation of multiple clones of B lymphocytes [12,35]. Moreover, it has been shown that epitopes have a high degree of mobility; thus, flexibility is also considered a good antigenic determinant. However, the inapplicable regions probably responsible for the antigenicity of the Cμ_1_ protein were not observed within the molecule, which suggests that these structural features of the protein could act as good epitopes by initiating innate and subsequently adaptive immune responses [36,37]. This protein molecule could potentially be considered suitable for protein expression and the further development of an applicable mAb.

Then, we successfully expressed a soluble IgMHCμ_1_ protein in the *E. coli* system. The IgMHCμ_1_ mAb against the IgM heavy chain of catfish was also potentially produced and developed using a rabbit model. Competitive ELISA and Western blot analyses were performed to exemplify the specificity and antigenic relatedness of the IgMHCμ_1_ mAb to the IgM heavy chain molecules of catfish as direct or cross-reactivity patterns of mAb IgMHCμ_1_ activity. In our results, the cross-reactivity of the IgMHCμ_1_ mAb was observed in African catfish and hybrid catfish but not in another distantly-related species, zebrafish, at any of the tested dilutions. Our Western blot and indirect immunofluorescence results suggest that the antigenic determinant of the IgMHCμ_1_ mAb in bighead catfish could recognize the serum tetramer (>245 KDa), serum monomer (~180 KDa), and serum native unfolded-halfmer (~63 KDa) IgMH molecules in catfish. Although cross-reactivity was not observed in zebrafish, it may be observed with varying intensity in other closely phylogenetically- related species at the family or order levels. In particular, the evolutionary phylogenetic relationship between the orders Siluriformes (catfish) and Cypriniformes (zebrafish), which have distinct evolutionary lineages, most likely affects the patterns of amino acid substitutions occurring within the different lineages [38]. Furthermore, the amino acid sequence similarity of IgMHCμ_1_ supported the distinct evolutionary relationships between bighead catfish and all other tested fish, including African catfish (82.4%), the hybrid (100%), and zebrafish (38.6%).

In addition, the IgMHCμ_1_ mAb could successfully identify IgM+ cells in catfish PBLs by the IIFAT method. The results demonstrated that specific fluorescence signals were observed in both the cell membrane and intracellular regions of the lymphocyte-like cells of all tested catfish. Two cell types, one with small, round cells with little cytoplasmic space and one with ellipsoid cells with large cytoplasmic regions, were found to exhibit an obvious positive reaction to the IgMHCμ_1_ mAb, indicating the specific characteristics of circulating B cells and plasma B cells, respectively. In this study, catfish lymphocytes were fixed with formalin fixative solution prior to the IIFAT assay, which alters the permeability properties of the membrane of IgM^+^ cells and eventually allows IgMHCμ_1_ mAb molecules to migrate to the cells. Additionally, the IIFAT method appears to show intracellular staining, but more research is required to conclusively demonstrate this. These results indicated that the IgMHCμ_1_ mAb could be applicable for identifying IgM^+^ cells in *Clarias* fish as well as in many previously-reported teleost fish [17,22,39,40,41,42,43,44,45,46]. It could suggest that these catfish possess B lymphocyte populations in their immune systems, as reported in other vertebrates [13,35].

The percentage of IgM^+^ cells in the PBLs of catfish was successfully investigated by flow cytometry using the IgMHCμ_1_ mAb. The catfish lymphocytes were gated, and the target B lymphocyte population was indicated by low Forward Scatter (FSC) and Side Scatter (SSC) scores in the dot plot Fluorescence-activated Cell Sorting (FACS) analysis [47]. Our results showed that the percentages of IgM^+^ cells of the healthy bighead catfish, African catfish, and their hybrid were 38.0–39.9%, 45.6–53.2%, and 58.7–60.0%, respectively. Although the IgMHCμ_1_ mAb was developed and was specific to the bighead catfish IgMHCμ_1_ protein, the lowest percentages of IgM^+^ cells were observed in bighead catfish. This result suggests that the percentages of lymphocytes have wide variations that also affect lymphocyte levels based on factors such as species characteristics and environmental and physiological factors [18,45]. The low percentages of IgM+ cells in bighead catfish in our results could probably result from its characteristics, such as natural susceptibility to particular infectious pathogens compared to the more resistant species, African catfish and the hybrid catfish. Moreover, previous studies also tested their mAbs by quantifying IgM^+^ cells in various economically-important and model species, including *P. olivaceus* (40.48%), *O. mykiss* (35–51%), *S. salar* (33%), and *D. labrax* (21%) [22,23,24,25].

In addition, according to our results, the IgMHCμ_1_ mAb could likely react with two major populations of IgM^+^ cells, recognizing both the soluble and membrane forms at the different stages of B lymphocytes. IgM is expressed intracellularly during the early stages of immature B cells and is expressed on the surface of mature B cells or secreted to the periphery. Nevertheless, the IgMHCμ_1_ mAb could be shown to react with other Ig isotypes in teleost fish, especially with IgD. At the molecular level, the teleost IgD molecule differs from its mammalian counterpart because the chimeric IgD protein contains a Cμ_1_ domain followed by a number of C_δ_ domains. This chimeric structure has also been reported in various teleost species, including Atlantic cod (*G. morhua*), Atlantic halibut (*Hippoglossus hippoglossus*), Atlantic salmon (*S. salar*), fugu (*Takifugu rubripes*), Japanese flounder (*P. olivaceus*), rainbow trout (*O. mykiss*), stickleback (*Gasterosteus aculeatus*), and zebrafish (*D. rerio*) [10,11,32,48]. To date, no complete fish IgD heavy chain without Cμ_1_ has been described. Although negative cross-reactivity within two major immunoglobulin isotypes, IgM and IgD, probably occurs in teleosts, it does not have severe adverse effects on any immunological parameter. IgD is sometimes said to be a mysterious immunoglobulin class in fish and other vertebrates [49,50]. First, it mainly appears on the surface early in B cells in identified vertebrates (except mammals and channel catfish (*Ictalurus punctatus),* in which it is found in both surface-bound and secreted forms) [50,51]. Second, the expression level of IgD is very low, approximately 3 μg/mL [51,52]. It is curious that no specific antibodies to serum IgD have been found in various conditions, including immunization or direct exposure to specific target antigens. In addition, the function of IgD has remained unclear, and specific tools for detecting IgD have not been developed to date [49,51,52,53].

Our findings revealed that the developed IgMHCμ_1_ mAb showed both high sensitivity and specificity to the target IgMHCμ_1_ protein in all of the tested immunological assays in catfish compared to previously-reported mAbs of teleosts [17,19,21,22,39]. These properties minimize the possibility of false negative and false positive signals among the obtained results. Furthermore, the developed IgMHCμ_1_ mAb can be successfully used as an immunological tool for the detection and quantification of IgM^+^ cells in catfish.

## 4. Materials and Methods

### 4.1. Animals

Bighead catfish (*C. macrocephalus* Günther, 1864), African catfish (*C. gariepinus*), and hybrid catfish (*C. macrocephalus* x *C. gariepinus*) weighing 90–120 g and zebrafish (*Danio rerio*) weighing 1.0–1.5 g were obtained from the Department of Aquaculture, Faculty of Fisheries, Kasetsart University, Thailand. They were acclimatized in a quarantine tank with aerated freshwater at temperatures between 28 and 31 °C for 2 weeks before the experiment. The experimental procedures performed with aquatic animals were carried out in accordance with the Ethical Principles and Guidelines for the Use of Animals recommended by the National Research Council of Thailand for the care and use of animals for scientific purposes. The protocol was approved by the Animal Ethics Committee, Kasetsart University, Thailand (Ethics ID: ACKU61-FIS-004).

### 4.2. Isolation of Peripheral Blood Leukocytes (PBLs)

Whole blood was drawn from the caudal vein of healthy bighead catfish, African catfish and hybrid catfish, and blood was collected from zebrafish (*Danio rerio*) by tail ablation with 0.1% (w/v) heparin. The blood was diluted 1:2 in RPMI-1640 (Gibco, Gaithersburg, MD, USA). The peripheral blood leukocytes (PBLs) were separated from other blood components using the Ficoll method [54]. Briefly, the blood cell suspensions were carefully laid over a discontinuous Ficoll solution (Histopaque®-1077, Sigma-Aldrich, Selangor, Malaysia) in a new sterile Falcon tube (the Ficoll:diluted blood ratio was 3:1). The tubes were centrifuged at 400 × g for 30 minutes. The white portion of the PBLs was collected and resuspended in 1.5 mL of RPMI [20,55].

### 4.3. Isolation of RNA and cDNA Synthesis

Total RNA was isolated from the PBLs of bighead catfish using NucleoZOL^TM^ reagent (Clontech Laboratories, Mountain View, CA, USA) according to the manufacturer’s instructions. The obtained total RNA was then used as the template for first-strand cDNA synthesis using a protocol described for the Thermo Scientific RevertAid Reverse Transcriptase Kit (Thermo Fisher Scientific, Waltham, MA, USA). The first-strand cDNA synthesis products were stored at −80 °C for further experiments.

### 4.4. Specific Primer Design and Amplification of cDNAs Encoding Cμ_1_ of the IgM Heavy Chain Protein

The specific primers used to obtain cDNA sequences encoding Cμ_1_ of the IgM heavy chain gene in bighead catfish were designed based on our previous study using completely-characterized full-length cDNA sequences of the IgM heavy chain (accession number MN652881). The *Nde* I (CA↓TATG) and *Xho* I (C↓TCGAG) specific restriction enzyme sites were added upstream of the 5′ end of the forward and reverse primers, respectively. The specific primers were *mAb_Cμ_1_-f*: 5′-CATATGGCAACACAAGAAGCCCCGAAGTCC-3′ and *mAb_Cμ_1_-r*: 5′-CTCGAGCAGCTCTG CCGTTTTCGTTCCCC-3′. The cDNA sequences encoding Cμ_1_ of the IgM heavy chain gene in bighead catfish were amplified using first-strand cDNA templates with the specific primers described above. PCR was performed using DreamTag DNA polymerase with proofreading activity (Thermo Fisher Scientific, Waltham, MA, USA) according to the manufacturer’s instructions. PCR was initiated with a predenaturation step at 95 °C for 5 minutes, followed by 30 cycles of 95 °C for 30 s, 55 °C for 30 s, and 72 °C for 2 minutes, and then finished with postextension at 72 °C for 10 minutes.

### 4.5. Cloning of the IgM Heavy Chain cDNA Sequences

The PCR products were purified from an agarose gel using the FavorPrepTM GEL/PCR Purification Kit (Favorgen Biotech, Ping-Tung, Taiwan) according to the manufacturer’s protocols as described previously. The nucleotides encoding Cμ1 of the IgM heavy chain protein of bighead catfish were first ligated to the pGEM^®^ T-Easy vector (Promega, San Luis Obispo, WI, USA), and then, the target DNA fragments were digested with the enzymes *Nde* I and *Xho* I. The digested DNA fragments were analyzed by gel electrophoresis and purified using the FavorPrep^TM^ GEL/PCR Purification Kit (Favorgen Biotech) according to the manufacturer’s protocols. Next, the purified DNA fragments with similar restriction enzyme sites were ligated to an *Nde* I-*Xho* I-digested pET-28b(+) expression vector (Thermo Fisher Scientific). The recombinant plasmids were transformed into the expression host *Escherichia coli* strain BL21 using heat and cold shock [56]. The plasmids from the positive recombinant clones were extracted using the FavorPrepTM Plasmid Extraction Mini Kit (Favorgen Biotech) according to the manufacturer’s protocols. The positive recombinant clones were analyzed using colony PCR screening and restriction enzyme digestion as described above. The positive uncut plasmids were verified by sequencing to confirm the insertion of the correct target sequences.

### 4.6. Antigenicity Characterization of the IgMHCμ_1_ Sequence of Bighead Catfish

The antigenicity of the protein encoded by the *IgMHCμ_1_* gene of bighead catfish was analyzed using the Optimum Antigen^TM^ design tool (GenScript, Piscataway, NJ, USA) according to the manufacturer’s instructions to determine the capacity of a biochemical structure to bind specifically with certain targets in adaptive immune components. Furthermore, the antigenicity positions of the IgMHCμ_1_ protein were predicted using programs integrated with the structural bioinformatics web-server SWISS-MODEL workspace (https://swissmodel.expasy.org) [56].

### 4.7. Expression and Induced Overexpression of Recombinant Protein

A single positive colony of a recombinant clone was inoculated into 20 mL of Luria-Bertani (LB) broth containing 30 μg/mL kanamycin and cultured in a shaking incubator at 37 °C for 18 hours. Then, 200 μL of the bacterial culture was transferred into 40 mL of LB broth containing 30 μg/mL kanamycin and grown at 37 °C with shaking at 160 rpm until the OD600 reached 0.6. For the induction of overexpression, isopropylthio-β-galactoside (IPTG) was added to the bacterial culture at a final concentration of 1.0 mM. Then, the bacterial culture was continuously incubated at 37 °C with shaking at 160 rpm for 5 hours. During the incubation period, at hours 0, 1, 2, 3, 4, and 5, 1 mL of bacterial solution was collected and centrifuged at 9,000 rpm for 5 minutes at 25 °C to optimize the protein expression conditions [57]. Recombinant protein expression in each of these fractions collected at different incubation times was analyzed using sodium dodecyl sulfate-polyacrylamide gel electrophoresis (SDS-PAGE) after incubating the samples with 2 × running buffer (Thermo Fisher Scientific) at 90–95 °C for 5 minutes. The supernatant was collected by centrifuging at 13,000 rpm for 3 minutes. Ten microliters of the supernatant and the appropriate molecular protein marker were loaded onto an SDS-PAGE gel and run at 100 volts for 10 minutes followed by 170 volts for 50 minutes. The gel was stained with Coomassie staining solution for 1 hour for expression analysis.

### 4.8. Protein Purification

The recombinant protein was purified with a HiTrap^TM^ Protein A HP column (GE Healthcare, Princeton, NJ, USA) using an ÄKTA pure protein purification system (ÄKTA pure 25 L, GE Healthcare) according to the manufacturer’s instructions. Briefly, 100 mL of bacterial culture was harvested by centrifugation at 5,000 rpm for 5 minutes. Cell pellets were resuspended in 10 mL of 20 mM Tris-HCl (pH 8.0) and disrupted by sonication on ice using six 20 s bursts at high intensity with a 20-second cooling period between each burst. The cell lysates were centrifuged at 12000 × g for 10 minutes at 4 °C to pellet the cellular debris. The supernatants were transferred to fresh tubes, and the remaining particles were removed by passing the sample through a 0.22 μm filter. Ten microliters of the cell pellets and supernatants was removed for SDS-PAGE analysis. Purification of target proteins was conducted using a HiTrap^TM^ Chelating HP 1 mL column (GE Healthcare) that was washed with distilled water. The column was equilibrated with 10 mL of 20 mM Tris-HCl, 0.5 M NaCl, 5 mM imidazole, 6 M guanidine hydrochloride, and 1 mM 2-mercaptoethanol (pH 8.0). The sample was loaded and washed with 10 mL of 20 mM Tris-HCl, 0.5 M NaCl, 5 mM imidazole, 6 M guanidine hydrochloride, and 1 mM 2-mercaptoethanol (pH 8.0). The column was washed with 10 mL of 20 mM Tris-HCl, 0.5 M NaCl, 20 mM imidazole, 1 mM 2-mercaptoethanol, and 6 M urea (pH 8.0). The recombinant protein was eluted using a 20 mL linear gradient starting with 20 mM Tris-HCl, 0.5 M NaCl, 20 mM imidazole, and 1 mM 2-mercaptoethanol (pH 8.0) and ending with the same buffer containing 500 mM imidazole. Ten microliters of each fraction were collected and analyzed by SDS-PAGE as previously described. Fractions containing the eluted protein were pooled and dialyzed to remove imidazole. The eluted protein was stored at −80 °C until use.

### 4.9. Production and Characterization of a Monoclonal Antibody (mAb) against the IgM Heavy Chain Protein

The purified protein encoding Cμ_1_ of the IgM heavy chain of bighead catfish was used to develop a mAb against the IgM heavy chain using GenScript’s MonoRab™ technology services (GenScript), according to the company’s procedures. Briefly, the highly purified Cμ_1_ of the IgM heavy chain protein with inorganic particulate aluminum salt as an adjuvant was injected into four New Zealand white rabbits for 8 weeks. The rabbits were immunized, and their blood was withdrawn over a 6-week period. This immunization step was repeated at 14 and 21 days after the first injection to boost the immune response (one primary injection and two booster injections) and further encouraged the development of mature, antibody-producing B cells. When the best-responding rabbits were boosted twice, their spleen cells were then harvested for hybridoma fusion (10 plates per fusion). B cells from the spleen of the target rabbits were then fused with immortal myeloma (HeLa) cells to generate hybridomas. The positive hybridoma cells were screened using ELISA. The positive cells were used to produce mAb in roller bottles to yield antibody-containing supernatants.

### 4.10. Characterization of Specific mAbs against the IgM Heavy Chain Protein of Catfish

#### 4.10.1. Indirect ELISA Analysis

Because the new mAb is specific to the IgM heavy chain of catfish, positive/negative threshold cutoff values were determined for optimization of the indirect ELISA. Initial reagent optimization was conducted using standard CBT as described in [58]. Bighead catfish and zebrafish sera were used as the positive and negative control sera. The coating antigen (purified catfish-IgMCμ_1_ protein) was titrated using dilutions ranging from 100 μg/mL to 0.09 μg/mL. Similarly, different serum dilutions ranging from 1:10 to 1:640 were tested. Serial dilutions of the newly-developed IgMHCμ_1_ mAb (1 mg/mL) ranging from 1:100 to 1:102,400 and of the secondary HRP-conjugated goat anti-rabbit IgG (1 mg/mL) ranging from 1:100 to 1:6,400 were also titrated. The optimum conditions were based on the optimum correlation of the linear plot of OD450 between positive and negative serum (P/N) OD values. A value close to 1.0 was considered optimal and selected for use in subsequent assays.

For competitive ELISA (c-ELISA), the optimum concentrations of serum and IgMHCμ_1_ mAb from the CBT assay were used. The microplate wells were firstly coated with 100 μL of purified Cμ_1_ protein as an antigen and incubated overnight at 4 °C. The wells were washed three times with phosphate-buffered saline (PBS) containing 0.05% Tween-20 (PBST; pH 7.4) and blocked with 100 μL of BlockPRO™ Blocking Buffer (Visual Protein, Taipei City, Taiwan) for 1 hour at 37 °C. During the washing period, the 100 μL of serial dilutions of test fish sera from 1:100 to 1:10,240 were preincubated with 100 μL of enzyme-labelled mAb IgMHCμ_1_ primary antibodies for 1 hour and then added mixture to the wells and incubated for 1 hour at 37 °C. The cELISA began when any of the unbound enzyme-labelled mAb IgMHCμ_1_ in the fish serum in the preincubation step binds to the coated recombinant Cμ_1_ antigen. After washing, the microplate wells were incubated with 100 μL of secondary HRP-conjugated goat anti-rabbit IgG (Sigma-Aldrich, St Louis, USA) diluted 1:12000 in PBS (pH 7.4) for 1 hour at 37 °C. After the last washing, 100 μL of TMB One Component HRP Microwell Substrate (Surmodics IVD, Inc., Eden Prairie, MN, USA) was added to each well, and the plates were incubated for 30 minutes at room temperature in the dark [45,59]. Then, the reaction was stopped with TMB Stop Solution (Surmodics IVD, Inc.). The absorbance of each reaction was measured with an iMark™ Microplate Absorbance Reader at 450 nm (Bio-Rad Laboratories Ltd., Hercules, CA, USA). The wells containing the mAb IgMHCμ_1_ negative c-ELISA reaction control and zebrafish sera were used as negative samples. Percentage competition was calculated using the following formula [45]:

%Competition = 100-[(OD of fish serum sample/OD of mAb control)]x100

#### 4.10.2. Western Blot Analysis

The IgM proteins in the sera of catfish and zebrafish were separated by 12% SDS-PAGE under native and reducing conditions. The diluted (1:100) sera samples were prepared in a buffer containing 100 mM Tris-HCl (pH 6.8) and 10% (w/v) SDS in the absence of 200 mM β-mercaptoethanol for native SDS-PAGE condition. The denatured sera proteins were incubated in a buffer containing 100 mM Tris-HCl (pH 6.8) and 10% (w/v) SDS in the presence of 200 mM β-mercaptoethanol at 100 °C for 15 minutes. Both native and denatured sera proteins were then electrophoretically transferred onto a polyvinylidene difluoride (PVDF) membrane (GE Healthcare). The membrane was blocked with BlockPRO™ Blocking Buffer (Visual Protein) for 1 hour at 37 °C. Then, the membrane was incubated with mAb IgMHCμ_1_, while zebrafish serum was used as the negative control. The membrane was washed three times with PBST (pH 7.4) and incubated with secondary HRP-conjugated goat anti-rabbit IgG (Sigma, USA), diluted 1:3000 in PBS (pH 7.4), and incubated at 37 °C for 1 hour. After the final wash, the membrane was stained with TMB One Component HRP Membrane Substrate (Surmodics IVD, Inc.), and staining was stopped by washing with distilled water. The reaction was considered positive upon detection of an insoluble permanent dark blue reaction product on the membrane. Zebrafish serum was used as a negative control [18,55].

#### 4.10.3. Indirect Immunofluorescent Assay Test (IIFAT) for the Detection and Quantification of IgM^+^ Cells

The PBLs of bighead catfish, African catfish, their hybrid, and zebrafish were prepared as previously described. The PBLs were counted and diluted to 10^8^ cell/mL and then were fixed with 10% v/v formalin fixative solution prior to the IIFAT assay, which alters the permeability properties of the membrane of IgM+ cells. The fixed PBLs were incubated with the IgMHCμ_1_ mAb for 1 hour at 37 °C. Subsequently, the cells were washed three times with PBS (pH 7.4) containing 0.05% (v/v) Tween solution, incubated with cross-adsorbed secondary antibody goat anti-rabbit IgG (H+L) conjugated with Alexa Fluor 488 (Invitrogen, Waltham, MA, USA) for 1 hour at 37 °C, and washed again. Then, the cell suspensions were placed on glass slides and observed by fluorescence microscopy with a Nikon C2 confocal microscope (Nikon, Melville, NY, USA). The PBLs of zebrafish were used as a negative control.

#### 4.10.4. Flow Cytometry Analysis for the Quantification of IgM^+^ Cells

The PBLs of each healthy catfish (*n* = 3) were isolated as described above, diluted to 1×10^7^ cells/mL, and fixed with 4% (v/v) paraformaldehyde at room temperature for 10 minutes. After washing twice with PBS (pH 7.4), the cells were pelleted by centrifugation at 2500 x rpm for 5 minutes. Subsequently, the PBLs were incubated with the IgMHCμ_1_ mAb for 1 hour at 37 °C. After washing twice with PBST (pH 7.4), the cross-adsorbed secondary antibody goat anti-rabbit IgG (H+L) conjugated with Alexa Fluor 488 (Invitrogen) was added, and the cells were incubated for 1 hour at 37 °C and then washed twice with PBST [20,45]. Positive IgM cells was quantified by flow cytometry (BD FACSCalibur, San Jose, CA, USA). PBLs without the IgMHCμ_1_ mAb were used as negative controls to optimize and standardize the system. The PBLs of zebrafish were used as negative fish samples.

## 5. Conclusions

A mAb specific to the IgM heavy chain of catfish was successfully developed using a rabbit model. The IgMHCμ1 mAb actively reacted with IgM of bighead catfish and cross-reacted with that of other related Clarias catfish, including African catfish and hybrid catfish. This IgMHCμ1 mAb could be a useful immunological tool for measuring catfish health status during cultivation. Furthermore, the knowledge obtained in this study can help us understand the specific immune system and development of B cells in fish immunity. In addition, it could help improve catfish aquaculture by selecting for specific disease resistance traits, helping prevent the dissemination of fish diseases and promoting the development of effective vaccines specific to catfish. These effects are beneficial for sustainable catfish aquaculture.

## Figures and Tables

**Table 1:** Percentage of IgM^+^ cells in peripheral blood leukocytes of teleost fish.

Fish	mAbs	IgM^+^ Cells (%)	References
Bighead catfish *(Clarias macrocephalus)*	IgMHCμ_1_ mAb IgMHCμ_1_ mAb IgMHCμ_1_ mAb	38.0–39.9	Present study Present study Present study
African catfish *(Clarias gariepinus)*		45.6–53.2	
Hybrid catfish *(C. macrocephalus x C. gariepinus)*		58.7–60.0	
Japanese halibut *(Paralichthys olivaceus)*	2D8	40.48	[22]
Rainbow trout *(Oncorhynchus mykiss)*	3D7, 4A8, 3E10,4C10	35–51	[23]
Atlantic salmon *(Salmo salar)*	Type II mAb	33	[24]
European bass *(Dicentrarchus labrax)*	DLIg3	21	[25]
